# Digital innovation for cancer risk assessment allows large-scale service redevelopment of regional cancer genetics service delivery

**DOI:** 10.1007/s10689-024-00407-x

**Published:** 2024-07-01

**Authors:** Alice Youngs, Andrea Forman, Marisa Elms, Kelly Kohut, Min Theik Hlaing, John Short, Helen Hanson, Katie Snape

**Affiliations:** 1 South West Thames Centre for Genomics, St George’s University Hospitals NHS Foundation Trust, SW17 0QT London, England; 2 Peninsula Clinical Genetics Service, Royal Devon University Healthcare NHS Foundation Trust, EX1 2ED Exeter, England; 3 St George’s University, London, UK

**Keywords:** Family history, Assessment, Digital, Cancer surveillance, Patient-facing resource

## Abstract

Family-history assessment can identify individuals above population-risk for cancer to enable targeted Screening, Prevention and Early Detection (SPED). The online patient-facing cancer Family History Questionnaire Service (cFHQS) is a digitalised, resource efficient tool for family history data capture to facilitate this. The capturing of digital data from cFHQS allows for data interrogation of patients referred to Clinical Genetics for the purposes of service improvement. Digital data from 4,044 cFHQS respondents over a three-year period was collected and interrogated with respect to the number and type of familial tumour diagnoses to enable service improvement and streamlining of referral pathways. 81% of colorectal and 71% of breast screening assessments were population- or moderate-risk. Most patients who completed cFHQS reported more than one diagnosis of cancer/tumour/polyps in their family. 2.5% of family history assessment patients had a second indication that required assessment that would have been missed if single tumour type assessment was undertaken. Implementation of an innovative, digital family history data collection pathway has allowed large scale interrogation of referral patterns and assessment outcomes to enable service development. The high volume of inappropriate referrals to Clinical Genetics for population and moderate risk patients highlighted the need for dedicated secondary care pathway provision for these patients. The use of cFHQS streamlined family history assessment allows for redistribution of resources to improve equity and access to genetic cancer risk assessment.

**Supplementary Information:**

The online version contains supplementary material available at 10.1007/s10689-024-00407-x.

## Background

Family history is a risk factor for developing health conditions including certain cancers such as breast and colorectal cancer [1, 2]. It is well documented that there are different hierarchies of genetic cancer risk and family history is a key influencer in stratifying risk to inform surveillance programmes for Screening, Prevention and Early Detection (SPED) [3]. The identification of genetically at-risk individuals for enhanced screening has been shown to reduce cancer mortality [4–6].

**Fig. 1:**
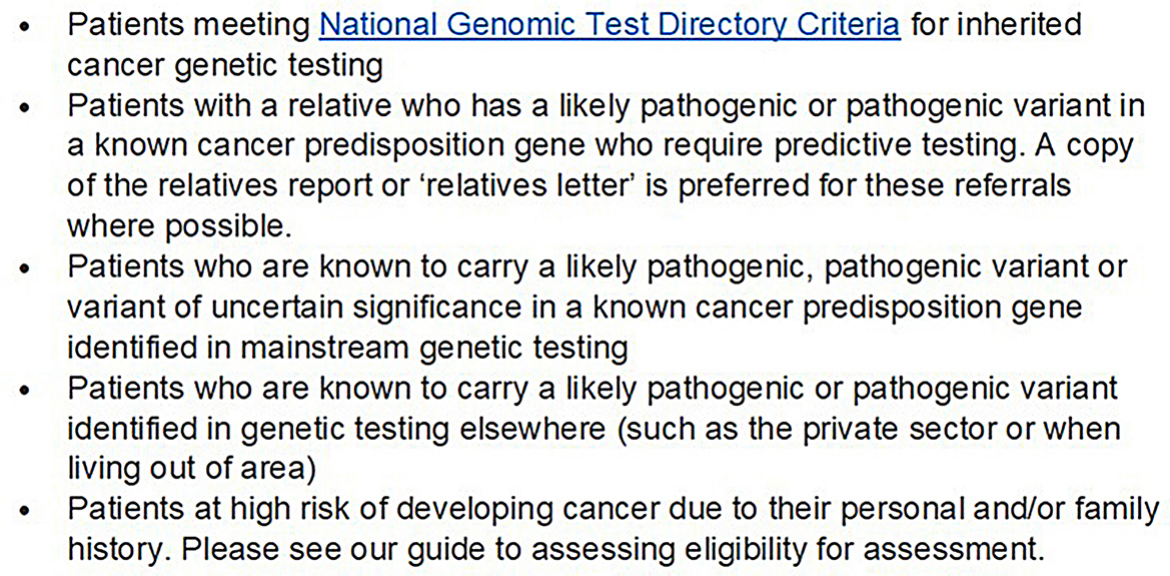
South West Thames Centre guidelines for Cancer Genetics referral [9]

**Fig. 2:**
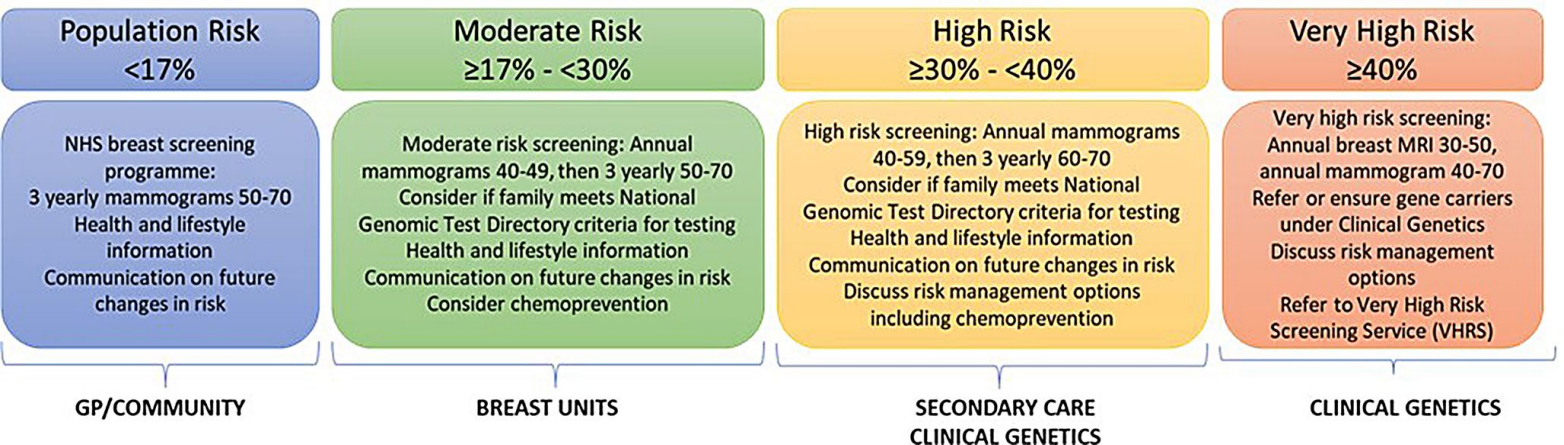
Exemplar of breast cancer stratified risk categories which map to different SPED interventions and different funding models across primary, secondary and tertiary care

The provision of family history assessment clinics is variable throughout the UK, with Clinical Genetics services seeing a rise in family history referrals [7]. Family history referrals often enter either already long Clinical Genetics waiting lists where predictive genetic testing for known high risk familial variants is needed, or symptomatic breast and endoscopy clinic waiting lists for which patients with current cancer require more urgent assessment [8]. Clinical Genetics are tertiary care services funded directly from NHS England via Specialised Commissioning to provide clinical services to those at high or very high genetic cancer risk, as well as other non-cancer related genetic conditions. The referral guidelines for the South West Thames Centre for Genomics (SWTCG) can be seen in Fig. 1. Patients at population risk for cancer should be managed by their GP and national cancer surveillance programmes and patients at moderate risk should be managed by secondary care, funded by regional Integrated Care Boards (ICBs) (Fig. 2).

Adherence to published National Institute for Health and Care Excellence (NICE) guidance for familial mammographic surveillance has been shown to be variable [7]. Assessment of family histories of cancer is generally tumour focused using published guidelines including NICE CG164 familial breast cancer [10], British Society of Gastroenterology (BSG) screening guidelines [11] and the National Genomic Test Directory [12]. Whilst published guidelines are helpful for underpinning surveillance, family history collection should allow for data capture that can be assessed across multiple guidelines to provide a pan-tumour assessment.

**Fig. 3:**
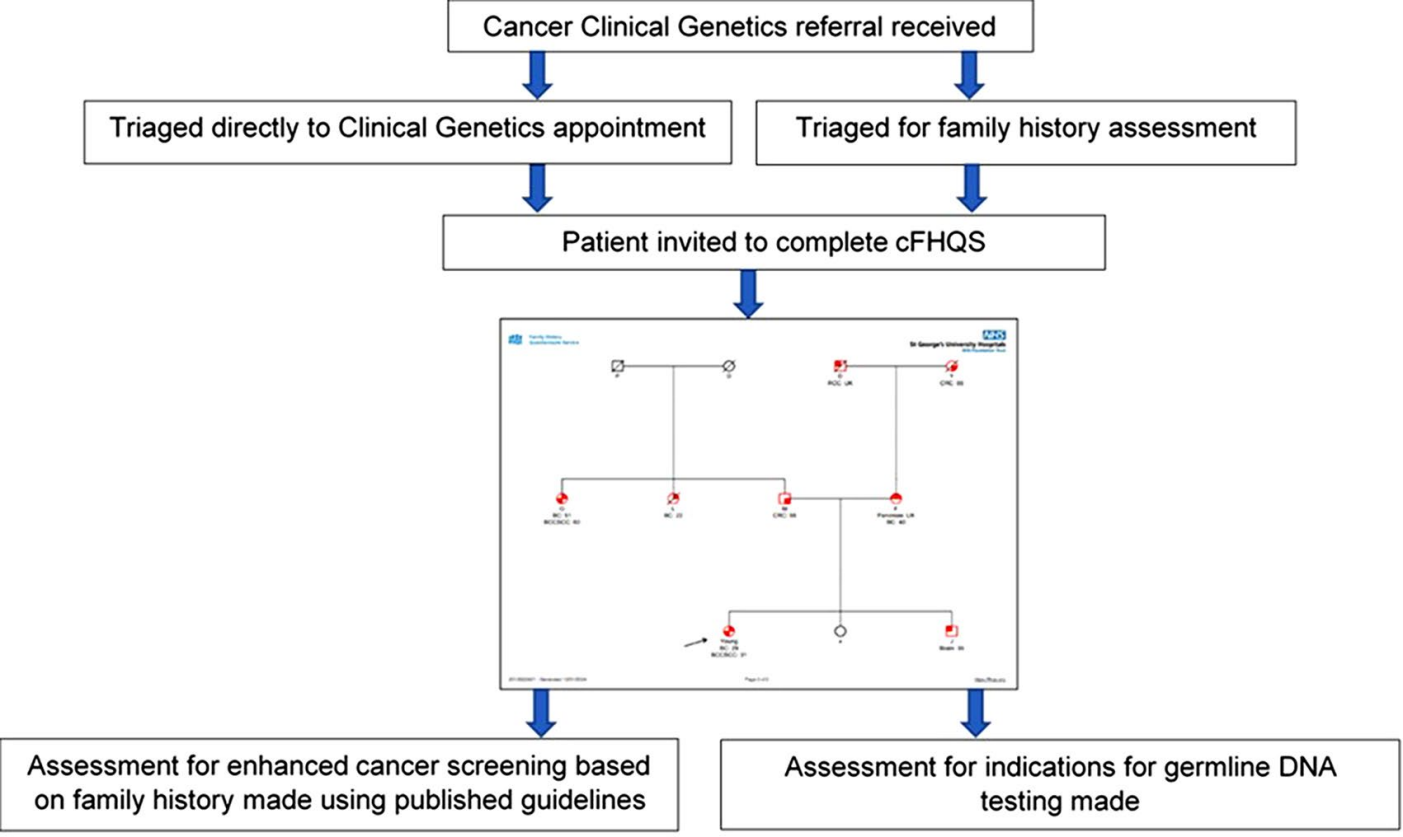
Exemplar of how cFHQS is used within the SWTCG. Where a referral is accepted into the service, patients are invited to complete cFHQS at home. The output of this is assessed against published guidelines and the National Genomic Test Directory [12]

To improve assessment of genetic cancer risk in the SWTCG region, we developed an online pathway to streamline the collection of family history information, termed the cancer Family History Questionnaire Service (cFHQS) [13]. The use of this online, patient-facing tool has facilitated the Clinical Genetics service to capture family history information prior to Clinical Genetics consultations with the additional benefit of being time effective, and acceptable to patients with respondents finding the tool easy to use and with a preference to the online input [14]. The output for cFHQS can help in the assessment of eligibility for germline DNA testing and support the assessment for enhanced cancer surveillance (Table 1; Fig. 3). The cFHQS tool has also been used as a low-resource tool in primary care pathways [15]. The output of cFHQS is compatible with the risk assessment tool CanRisk [16–18], easing the manual burden of CanRisk [16–18] assessment for lifetime breast and ovarian cancer risks for unaffected individuals but also for other indications, for example in assessing indications for germline DNA testing in affected patients as well as guide initiation of breast screening in *BRCA1, BRCA2* and *PALB2* gene carriers.

**Table 1:**
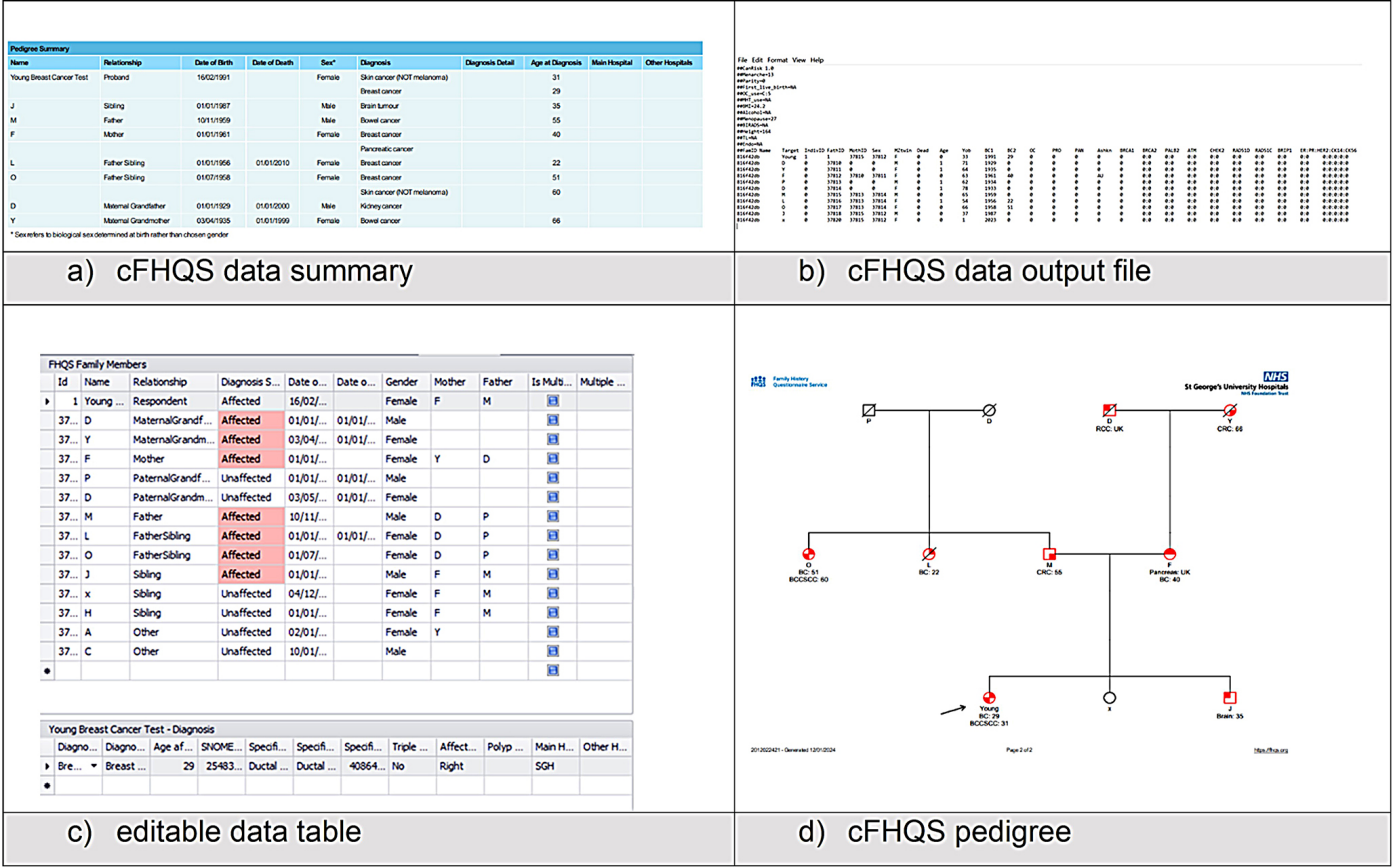
Diagram showing example outputs of cFHQS. (a) Family history data summary (b) The data output file (c) The editable family history data table and (d) The pedigree created by cFHQS

Traditionally, Clinical Genetics Services have obtained family history information on either written paper questionnaires or pedigree drawing in clinic. Implementation of cFHQS for digital family history data capture had the additional advantage of allowing data interrogation for the purposes of service development. We aim to demonstrate how digital family history data captured by cFHQS has been used to assess referral patterns, tumour types in families and improve delivery of cancer genetics risk assessment in our region. We used the digital data from cFHQS to consider three key questions:

1. Are we equally serving our population of patients in terms of diversity and access?
2. Are we providing an appropriate service to our region for the funding that we receive in Clinical Genetics?
3. Are we enabling all patients to receive any genomic testing or SPED they are eligible for across tumour types?

## Materials and methods

### Patient series

We included data from all patients who completed cFHQS between 01/10/2019 and 30/09/2022 regardless of indication for referral. Data were taken directly from the cFHQS website and compared with those on the Clinical Genetics internal database ‘GeneWorks’. This dataset included family history, diagnostic and predictive testing referrals. Not all referred patients completed cFHQS as a significant proportion of those who were triaged directly into appointments gave their family history manually in the appointment. This means the cohort is enriched for those who required family history assessment for triage purposes compared with those referred directly for diagnostic or predictive genetic testing.

### Demographic analysis

Ethnicity and gender data were self-reported by patients in the cFHQS dataset. These were compared with the 2021 regional census data [19, 20]. Ethnicity census data was obtained for each of the wards covered by the SWTCG and an average for each ethnic group was calculated.

### Assessment outcome

Clinician-inputted diagnostic coding on Geneworks was used to identify those patients who had received a family history assessment outcome including those who had received a breast cancer screening outcome, a colorectal cancer screening outcome, or been given an additional diagnosis related to their personal or family history. We considered that referrals appropriate for Clinical Genetics would have a final assessment of either high or very high risk. Referrals at moderate or population risk were deemed inappropriate for referral to a tertiary regional genetics service. Breast screening assessments in this period were undertaken using the Institute of Cancer Research Protocol 1 [21] to map breast screening recommendations to current NICE CG164 guidelines [10]. Colorectal cancer screening assessments were undertaken using the 2019 BSG Guidelines [11]. Assessments of eligibility for germline genetic testing was undertaken according to the National Genomics Test Directory [12].

## Results

From 1st October 2019-30th September 2022, 4,044 patients completed cFHQS. The average time from the patient registration with the online cFHQS system to submission of the completed form was 5.41 days. Outcomes with respect to the three research questions are reported below.

### (1) Are we equally serving our population of patients in terms of diversity and access?

The average age of the patients with a completed cFHQS was 47 years old (3–92 years).

2,654/4,044 (65.6%) patients reported their ethnicity as white British, 573/ 4,044 (14.2%) as any other white Background and 127/4,044 (3.1%) as white Irish. White British, any other white background and white Irish were all overrepresented when compared with 2021 census ethnicity data for the SWTCG region [19]. 76/4,044 (1.9%) of respondents were from an Indian background, 44/4,044 (1.0%) Pakistani background, 52/4,044 (1.3%) from a black Caribbean background and 40/4,044 (0.99%) from a black African background. These ethnic minority groups, amongst others were all underrepresented when compared with 2021 census data (Table 2).

**Table 2:** Ethnicity data for the 14 most reported ethnicities compared with 2021 census data

Ethnicity	Total number of patients reporting this ethnicity	Ethnicity as a percentage	Census data from the SWTCG region
White British	2654	65.6%	56.9%
Any Other White Background	573	14.2%	5.2%
White Irish	127	3.1%	1.5%
Indian	76	1.9%	4.2%
Any Other Mixed Background	58	1.4%	1.6%
Any Other Asian Background	57	1.4%	4.4%
Mixed White And Asian	53	1.3%	1.5%
Black Caribbean	52	1.3%	2.7%
Pakistani	44	1.0%	2.9%
Black African	40	0.9%	4.1%
Chinese	35	0.8%	1.4%
Mixed White And Black Caribbean	16	0.4%	1.3%
Mixed White And Black African	14	0.3%	0.7%
Any Other Ethnic Group	13	0.3%	2.6%

Data on sex and gender demographics showed that 3,359/4,044 (83%) of the individuals who completed cFHQS reported being assigned female at birth and 685/4,044 (17%) reported being assigned male at birth. 15/4,044 (0.37%) provided a gender identity that differed with the sex assigned at birth. The percentage of patients with gender diversity was lower than expected in the SWTCG region which is estimated to be 0.5% [20].

### (2) Are we providing an appropriate service to our region for the funding that we receive in Clinical Genetics?

1,349/4,044 (33%) patients reported a personal cancer/tumour/polyp diagnosis. The most common reported personal diagnosis was breast cancer 449/1,349 (33%) (supplementary Table 1). The remaining 2,695/4,044 (67%) patients who completed cFHQS were unaffected with cancer.

We considered only the 2,695 unaffected individuals with respect to final assessment outcome and appropriateness of referral.

After accounting for duplicate diagnostic codes, 1,376/4,044 (34%) patients who completed cFHQS were assigned a breast or colorectal cancer screening status diagnostic code (Table 3). 277/1,376 (20%) were colorectal screening codes and 1,099/ 1,376 (80%) were breast screening codes. 224/277 (81%) of colorectal screening recommendations were population or moderate risk. 785/1,099 (71%) of breast screening recommendations were population or moderate risk.

**Table 3:** Screening status for unaffected patients who completed cFHQS

Breast screening assessment	Number of patients	Colorectal screening assessment	Number of patients
Population risk	321/1,099 (29%)	Population risk	81/277 (29%)
Moderate risk	464/1,099 (42%)	Moderate risk	143/277 (52%)
High risk	310/1,099 (28%)	High risk	40/277 (14%)
Very high risk	4/1,099 (0.36%)	High risk (Amsterdam positive)	13/277 (5%)

### Are we enabling all patients to receive any genomic testing or SPED they are eligible for across tumour types?

36 different cancer/tumour/polyp types were reported across the 4,044 patients, excluding “other” free text options. 3,566/4,044 (88%) patients reported more than one cancer/tumour/polyp diagnosis in their family. The average number of different cancer/tumour/polyp diagnoses in the pedigrees of the 4,044 patients who completed cFHQS was 4 (0–34).

Geneworks was reviewed for second diagnosis codes to assess if multiple pan-tumour recommendations were made based on the family history provided for this cohort. Of the 1,376 patients with a screening diagnosis code, 402 were population risk (29%). After removal of population risk patients, 12 out of 974 (1.2%) received a screening diagnosis code for both breast and colorectal cancer.

62/974 (6%) patients had a screening status code for breast or colorectal cancer and another unrelated cancer diagnosis coded on Geneworks. 13/974 (1.3%) required action because of the second diagnosis. When adding together those patients with both a breast and colorectal screening diagnosis and those with an actionable second cancer diagnostic code, 25/974 (2.5%) patients would have missed out on a second screening or genetics recommendation if the assessment was focused on a single cancer type alone.

In addition, 2/974 (0.2%) patients were referred back to Clinical Genetics following the original screening assessment with a new cancer genetic diagnosis and were eligible for genetic testing. 2/974 (2%) patients were offered further investigation by the rare disease arm of Clinical Genetics team due to a rare disease indication noted on the cFHQS summary. Indications included motor neurone disease (MND) and premature ovarian failure.

## Discussion

Our novel digital pathway for family history data collection enables large scale interrogation of family history data alongside demographic information. We explored three key questions for the purposes of improving delivery of cancer genetics services across our region.

### (1) Are we equally serving our population of patients in terms of diversity and access?

The data presented here shows that we are underserving patients of non-white ethnicity in our region, when compared with the ethnicity census data. This has previously been reported in the literature with a study in 2001 finding that only 3% of UK cancer genetics referrals were from individuals of ethnic minority groups [22]. Discrepancies in access to healthcare for those from ethnic minority groups is well documented and could be related to low awareness of familial cancer or belief systems [23] amongst other unknown cultural factors and barriers. Our cFHQS assessment pathway enables streamlined family history assessment for those who are able to complete assessments independently. We are now developing pathways for additional administrative and clinical resource to look at non-responders and to arrange telephone calls to support those who have not provided data in response to their referral. We are undertaking further work to look at how this redistribution of resource may improve uptake of assessment in those with non-white ethnicity and other underserved groups. In addition, saved clinical time can be prioritised for patients with greater access needs including language and digital literacy.

The age range of individuals completing cFHQS was wide. cFHQS allows parents and guardians to input family history information for children referred with a personal history of paediatric tumours to enable assessment of the family history against current germline genetic testing eligibility. The cFHQS tool allows the capture of a wide range of tumour types including paediatric tumours.

cFHQS offered a selection of choices for gender identification which allowed 15 patients (0.37%) to express gender diversity. However, the number of patients with gender diversity was lower than expected compared with census data for the SWTCG region which estimates this to be approximately 0.5% [20]. Documented barriers to these patients accessing specialist services include lack of onward referral or concern about being given incorrect advice [24]. We are undertaking further work to consider the needs of this population within our service.

Future work will be undertaken to improve the uptake of cFHQS by patients whose first or preferred language is not English, through the translation of invitation letters and, working with trusted, local community groups to ensure cultural sensitivity. The accessibility of this online tool will continue to be evaluated to make it more relevant and inclusive to better meet the needs of our diverse patient population.

### Are we providing an appropriate service to our region for the funding that we receive in Clinical Genetics?

In the UK, NICE guidance CG164 for familial breast cancer recommends that a family history be taken in primary and secondary care, when a person presents with symptoms or concerns about relatives with breast cancer [10]. Those patients at high risk are eligible for a Clinical Genetics review.

The data presented here show that 81% of colorectal and 71% of breast screening assessments were population or moderate risk and highlighted the volume of unfunded work being undertaken by Clinical Genetics services. Previous studies explored the appropriateness of referrals to Clinical Genetics [25, 26]. One study found that only 43% of referrals to Clinical Genetics from primary and secondary care were above population risk [27]. This has also been studied outside of the UK with an American study reporting that 92% of a sample of referrals from primary physicians did not meet referral guidelines [28]. Most patients (67%) reviewed here were unaffected despite current National Genomic Test Directory [12] guidelines suggesting that it is those patients with cancer where genetic testing is more appropriate. Although not in use at the time of this review, the use of cFHQS will allow for wider use of the risk assessment tool CanRisk [16–18].

There is a need for improvements in the care of family history patients, both in the recording of family history of cancer [29] and the implementation of NICE guidance for familial mammographic surveillance which has been shown to be variable across the UK [7]. The lack of funding for Clinical Genetics for population and moderate risk patients and the variable provision in the UK, highlights the need for funding to repatriate moderate risk patients out of Clinical Genetics and into a dedicated family history service.

We have now implemented an independently funded secondary care Virtual Family History Clinic (VFHC) within our region supported by funding from the RM Partners [30] and Surrey and Sussex Cancer Alliances [31] to address this need. This service provides family history assessments by secondary care family history nurses supported by the Clinical Genetics service to ensure the highest quality care for genetically at-risk individuals. Long term commissioning of this service from the ICBs will be required to ensure these patients receive sustainable care. Further work will assess the impact of the VFHC on reduction in inappropriate referrals, improved access to cancer risk assessment and the identification of more genetically at-risk individuals in the community in an inclusive manner.

### Are we ensuring patients are assessed across tumour types for all SPED and genomic testing they are eligible for?

Assessment of family history of cancer is generally tumour focused using published guidelines including NICE CG164 familial breast cancer [10], British Society of Gastroenterology (BSG) screening guidelines [11] and the National Genomic Test Directory [12]. Multifactorial risk models require accurate records relating to familial risk and reproductive factors [32].

In our data set, most patients who completed cFHQS reported more than one diagnosis of cancer/tumour/polyps in their family, with an average of four tumour types. This suggests the need for a pan-tumour assessment looking across multiple guidelines as opposed to assessment focussed on only one tumour type such as breast or colorectal.

1.2% of patients who had a family history assessment, had an enhanced screening recommendation for both breast and colorectal cancer, suggesting that looking at only one tumour type would not have been sufficient for this patient cohort. Additionally, 1.3% with a breast or colorectal screening assessment required input for a second unrelated diagnosis code from cFHQS. 2.5% of patients required a second assessment that would have been missed if single tumour type assessment was undertaken. This shows the value in cFHQS at capturing a range of tumour types to aid family history assessment and facilitate the use of multifactorial risk models. Whilst this proportion is relatively small, patients are unlikely to provide this level of granular detail on family history multiple times. Any new diagnoses in the family can also be added to previous risk assessments easily without needing to retake the whole history and perform a new calculation. Our findings proved pan tumour assessment adds value to this patient cohort.

## Summary

Limitations of the data presented include the reliance on clinicians to appropriately code patients on the Clinical Genetics Geneworks database when finalising the assessment. Some patients may not have received a diagnosis code, or this may have been added as an alternative diagnosis code on completion of the family history assessment. Therefore, this cohort is not entirely representative of all referred patients. However, the key findings of this work are directly applicable to service development plans.

Implementation of a digital family history data collection pathway has allowed large scale interrogation of our referral patterns and assessment outcomes to enable service development. The data presented here have supported the development of a Virtual Family History Clinic (VFHC), a dedicated nurse-led virtual family history clinic to improve the provision of family history assessments. Streamlining of this assessment pathway enables us to redistribute resource to under-served communities to improve equity and access to genetic cancer risk assessment.

## Conclusion

Ensuring resources in Clinical Genetics Services are appropriately directed to those at highest risk is challenging. The implementation of digital family history data capture has provided an opportunity to streamline this and has the added advantage of being compatible with risk assessment tools and provides the ability to perform large scale data interrogation. The high volume of inappropriate referrals to Clinical Genetics for population and moderate risk patients in our region highlighted the need for dedicated provision for these patients. Use of the streamlined cFHQS pathway for family history assessment also allows for resource reallocation to improve accessibility and uptake of cFHQS.

## Electronic supplementary material

Below is the link to the electronic supplementary material.

Supplementary Material 1

## Data Availability

No datasets were generated or analysed during the current study.
