# Supplementary material for: Digital innovation for cancer risk assessment allows large-scale service redevelopment of regional cancer genetics service delivery
Source: Fam Cancer. 2024 Jul 1;23(4):591–8. doi: 10.1007/s10689-024-00407-x (PMC11512827; doi:10.1007/s10689-024-00407-x)
Supplement: Supplementary file 1 — Supplementary Material 1 [file 10689_2024_407_MOESM1_ESM.docx]

| **Cancer types reported in FHQS** | **Total no. of FHQS Patients** |
| --- | --- |
| Breast cancer | 449 |
| Bowel polyps | 311 |
| Bowel cancer | 116 |
| Melanoma | 67 |
| Skin cancer (NOT melanoma) | 66 |
| Unknown | 57 |
| Prostate cancer | 45 |
| Womb cancer | 43 |
| Ovarian cancer | 28 |
| Brain tumour | 21 |
| Lymphoma | 20 |
| Thyroid cancer | 15 |
| Hyperparathyroidism | 15 |
| Leukaemia | 13 |
| Sarcoma | 10 |
| Bladder cancer | 10 |
| Kidney cancer | 9 |
| Lung cancer | 7 |
| Cervical cancer | 7 |
| Pituitary tumour | 5 |
| Spinal tumour | 4 |
| Paraganglioma | 4 |
| Testicular cancer | 3 |
| Phaeochromocytoma | 3 |
| Hypercalcaemia | 3 |
| Pancreatic cancer | 3 |
| Gastrointestinal stroma tumor | 2 |
| Wilms tumour | 2 |
| Liver cancer | 2 |
| Parathyroid adenoma | 2 |
| Multiple myeloma | 2 |
| Oesphageal cancer | 2 |
| Pancreatic neuroendocrine tumour | 1 |
| Mesothelioma | 1 |
| Stomach cancer | 1 |
| Urinary tract cancer | 1 |

**Supplementary material**

**Table 1- cancer types reported in respondents to cFHQS exclude “other” free text options**
